# Oxymatrine Alleviates Cerebral Ischemia/Reperfusion Injury By Targeting HDAC1 to Regulate Mitochondria-Related Autophagy and Oxidative Stress

**DOI:** 10.1007/s12035-025-05423-1

**Published:** 2025-11-29

**Authors:** Chang-Sheng Ma, Bo Han, Yu-Xi Liu, Chang-Ku Shi, Dong-Lun Li, Jin-Fen Guo, Min Bai, Shu-Chen Meng, Li-Ying Zhang, Meng-Yuan Duan, Mao-Tao He

**Affiliations:** 1 School of Basic Medical Sciences, Shandong Second Medical University, Weifang Shandong, 261053 China; 2 Department of Anesthesiology, Shanghai Key Laboratory of Maternal Fetal Medicine, Shanghai First Maternity and Infant Hospital, School of Medicine, Shanghai Institute of Maternal-Fetal Medicine and Gynecologic Oncology, Tongji University, Shanghai, 200092 China; 3 Affiliated Hospital of Shandong Second Medical University, Weifang Shandong, 261053 China

**Keywords:** Oxymatrine, Cerebral ischemia/reperfusion (I/R) injury, HDAC1, Excessive autophagy, Oxidative stress

## Abstract

Oxymatrine (OMT), a major alkaloid extracted from Sophora flavescens, has been widely recognized for its anti-inflammatory and anti-cancer properties. However, its precise neuroprotective mechanisms in cerebral ischemia/reperfusion (I/R) injury remain to be fully elucidated. In this study, we combined in vivo and in vitro models to investigate the therapeutic effects of OMT on cerebral I/R injury and glutamate-induced neuronal toxicity during the reperfusion process. In vivo, a mouse middle cerebral artery occlusion (MCAO) model was established to recapitulate I/R injury, whereas glutamate-exposed HT22 hippocampal neurons were utilized as an in vitro model to mimic excitotoxic damage. Bioinformatics analysis, integrated with molecular docking, identified histone deacetylase 1 (HDAC1) as a potential direct target of OMT. Subsequent experimental validation demonstrated that OMT attenuates I/R-induced brain damage by modulating HDAC1-mediated pathways involved in autophagy and oxidative stress regulation. OMT treatment significantly reduced infarct volume and improved neurological function in mice. At the cellular level, OMT suppressed mitochondrial apoptosis and reactive oxygen species (ROS) accumulation, restored mitochondrial membrane integrity, and rebalanced mitochondrial dynamics by downregulating fission-related proteins (Fis1) and upregulating fusion markers (Mfn2). Additionally, OMT inhibited excessive autophagy through modulation of the PINK1/Parkin signaling pathway, as evidenced by decreased expression of LC3-II/I ratio, PINK1, Parkin, and NBR1, along with restored levels of P62.These findings suggest that OMT exerts its neuroprotective effects in cerebral I/R injury by targeting HDAC1, thereby alleviating oxidative stress and excessive autophagy. This study provides new mechanistic insights and supports OMT as a promising therapeutic candidate for ischemic stroke treatment.

**Graphical Abstract:**

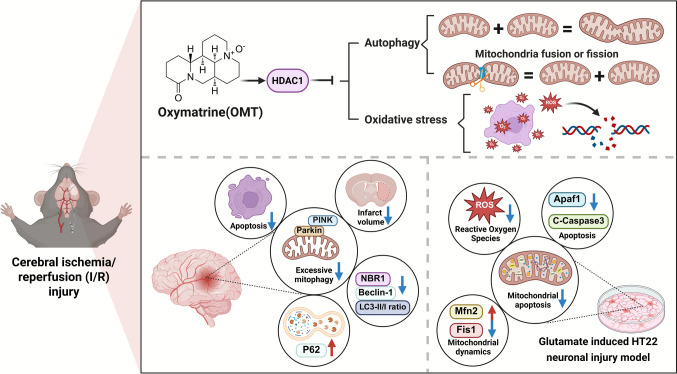

## Introduction

Ischemic stroke (IS) represents the most common subtype of cerebrovascular disease, characterized by its high rates of morbidity and disability, accounting for approximately 80% of all stroke cases [1, 2]. Cerebral I/R injury occurs as a secondary injury during cerebral blood flow reperfusion [3]. It has been shown that cerebral I/R injury leads to brain tissue damage, inflammation, mitochondrial dysfunction, apoptosis, and oxidative damage [4, 5]. Unfortunately, the current effective treatments are limited [6, 7]. Consequently, there is an urgent need to explore alternative preventive and treatment strategies, particularly those involving neuroprotective agents [8].

Sophora flavescens is indeed used in traditional Chinese medicine, where it’s dried; the root and flowers are believed to have medicinal properties [9]. The plant contains active compounds, including alkaloids, that have been found to have anti-cancer, anti-inflammatory, and other potentially beneficial activities [10]. These properties have made Sophora flavescens an important ingredient in traditional medicine and have also attracted the attention of scientific research into potential pharmaceutical applications. Matrine, oxymatrine, sophorocarpine, sophoridine, and oxymatrine from Sophora flavescens are potent bioactive compounds. Among them, OMT is one of the main bioactive ingredients extracted from the root of Sophora flavescens. It is a natural quinoline alkaloid compound from Sophora flavescens. Studies have confirmed that oxymatrine has a variety of pharmacological properties, including anti-oxidative effects, anti-inflammatory effects, and anti-proliferative effects [11–13]. According to our previous studies, oxymatrine might protected microvessel in the brain [14]. In addition, oxymatrine reduces brain infarct volume and edema in a cerebral ischemia and reperfusion model [15]. Hence, oxymatrine has considerable potential for development and utilization in the future, such as health food or functional food additives. However, the effects of oxymatrine on mitochondrial autophagy and mitochondrial dynamics are not well understood [16].

Mitochondrial autophagy (mitophagy) in concert with mitochondrial dynamics (fusion and fission) sustains mitochondrial homeostasis and plays an essential role in cerebral I/R injury. In mitochondria, the term “mitochondrial dynamics” refers to the delicate equilibrium between fusion and fission processes that preserve mitochondrial shape, distribution, and size. Mitochondrial fission is promoted by factors such as fission protein 1 (Fis1), while mitochondrial fusion is carried out by proteins such as Mfn1, Mfn2 [17]. It is well known that mitochondrial function is strongly related to mitochondrial dynamics [18]. Furthermore, an imbalance in mitochondrial dynamics may trigger autophagy [19]. Over activation of mitochondrial autophagy may degrade mitochondria, leading to autophagic cell death. Additionally, mitochondrial autophagy may promote apoptosis, generate additional ROS, and degrade mitochondria via the PINK1/Parkin pathway [20]. Upon the onset of mitochondrial stress, PINK1 and Parkin were recruited to the mitochondria to initiate PINK1/Parkin pathway-dependent mitochondrial autophagy [21].

This study investigated the neuroprotective effects of OMT in cerebral I/R injury using in vivo and in vitro models. A mouse MCAO model and glutamate-induced HT22 neuronal injury model were employed. Key markers of mitophagy (PINK1, Parkin, LC3-II/I, P62) and mitochondrial dynamics (Fis1, Mfn2) were assessed by western blot and immunofluorescence. Bioinformatics and molecular docking analyses identified HDAC1 as a target of OMT. Functional experiments revealed that OMT protects neurons by promoting HDAC1, reducing excessive autophagy through the PINK1/Parkin pathway, restoring mitochondrial dynamics, and alleviating oxidative stress. These findings suggest OMT as a potential neuroprotective agent with applications in nutraceuticals for I/R injury.

## Materials and Methods

### Animal Experiments and Methods

In this study, we employed 164 male C57BL/6 mice, aged between 6 and 8 weeks. The experimental procedures received approval from the Institutional Animal Care and Use Committee and the Ethics Review Committee of Shandong Second Medical University (2021SDL481). The animals were housed in the Laboratory Animal Center of Shandong Second Medical University under controlled conditions: a temperature range of 20 − 23 °C, humidity levels of 40 − 70%, and a 12-h light/dark cycle. The mice were randomly assigned to three groups: the Sham group, the I/R group, the I/R+OMT group (administered oxymatrine intraperitoneally at a dosage of 100 mg/kg/day for one week before MCAO and during the reperfusion period), and the I/R+OMT + Vorinostat group, treated with oxymatrine (as above) combined with Vorinostat (SAHA, MCE, China,100 mg/kg in 200 μl PBS, administered intraperitoneally once daily for 3 weeks) [22].

The cerebral I/R injury model was established using the MCAO method as traditional. Briefly, mice were anesthetized with 2% isoflurane for induction, maintaining anesthesia with 1.5% isoflurane during the procedure. A monofilament (Doccol Corporation, USA) was inserted via the common carotid artery to obstruct the middle cerebral artery. The filament was removed to restore blood flow after 60 min of occlusion. Neurological function was assessed 24 h after reperfusion using the modified neurological severity score (mNSS) system.

### Cell Culture and Treatment

HT22 cells were cultured in DMEM/F12 medium (HyClone Laboratories, Waltham, MA) supplemented with 10% fetal bovine serum (FBS) and 1% penicillin/streptomycin (Gibco, USA) under standard conditions (37 °C, 5% CO₂, 90% humidity), with medium replacement every 24 h. To induce oxidative injury, cells were treated with glutamate at graded concentrations (2, 4, 6, 8, or 10 μM) for 24 h. Concurrently, oxymatrine (5, 15, 35, or 45 μM) was administered to assess neuroprotective effects. Control groups included untreated cells (fresh medium only) and vehicle controls (equivalent volumes of PBS for glutamate or ≤0.1% DMSO for OMT). HT-22 cells were seeded in 96-well plates at a density of 5 × 10^3^ cells/well and allowed to adhere for 24 h under standard culture conditions (37 °C, 5% CO₂). After treatment, 10% (v/v) CCK-8 reagent (BS350B, Biosharp, China) was added to each well, followed by incubation at 37 °C for 3 h. Absorbance was measured at 450 nm using a Multiskan FC microplate reader (Thermo Fisher Scientific, Shanghai, China).

### Measurement of MnSOD Expression and Intracellular Superoxide Levels

The expression of manganese superoxide dismutase (MnSOD) and intracellular superoxide levels in HT-22 cells was analyzed via Western blot and DHE fluorescence staining, respectively. For MnSOD detection, cells were lysed in RIPA buffer (P0013, Beyotime, China) containing protease inhibitors (A32963, Thermo Fisher, USA). Protein concentrations were quantified using a BCA assay kit (BL521A, Biosharp, China), and 30 μg of total protein per sample was separated by 12% SDS-PAGE. Membranes were blocked with 5% non-fat milk and incubated overnight at 4 °C with a primary antibody against MnSOD (1:1000, ab13533, Abcam, UK), followed by HRP-conjugated secondary antibody (1:5000, SA00001-2, Proteintech, China) at room temperature for 1 h. Signals were visualized using an ECL substrate (BL520A, Biosharp, China) and quantified via ImageJ.

For superoxide detection, cells were incubated with 10 μM dihydroethidium (DHE, S0063, Beyotime, China) in serum-free medium at 37 °C for 30 min in the dark. After three PBS washes, fluorescence images were captured using a confocal microscope (Zeiss LSM 880, 543 nm excitation/590 nm emission) at 400 × magnification. Fluorescence intensity was normalized to cell count using ImageJ.

### Open Field Test

Locomotor activity and anxiety-like behavior were evaluated using an open field apparatus (40 × 40 × 30 cm, white acrylic chamber; RWD, China). Mice were individually placed in the center and allowed to freely explore for 10 min under dim light. Movement trajectories were recorded by an overhead camera and analyzed via ANY-maze software (Smart 3.0, RWD, China). Total distance traveled (mm) was quantified. The apparatus was cleaned with 70% ethanol between trials to eliminate odor cues.

### Y-Maze Novel Arm Exploration Test

Spatial novelty exploration was evaluated using a Y-maze composed of three identical arms (35 cm length × 10 cm width × 15 cm height; 120° inter-arm angles; Shanghai Jiliang, China). During the training phase, mice were permitted to explore two arms for 10 min, while the third arm (novel arm) was temporarily blocked. After a 24-h interval, the test phase was conducted by unblocking the novel arm and allowing free exploration of all three arms for 8 min. Arm entries were defined as the complete entry of all four paws into an arm. Exploration behavior was analyzed for three parameters: (1) novel arm entry preference (percentage of entries into the novel arm relative to total entries), (2) time spent in the novel arm (seconds), and (3) latency to first novel arm entry (time delay in seconds). Trials with fewer than 5 total arm entries were excluded to ensure behavioral validity. The maze was rigorously cleaned with 70% ethanol between trials to eliminate residual olfactory cues.

### Western Blotting

Tissue of ischemic penumbra of the cerebral cortex or cell samples were lysed, and protein concentrations were determined using the BCA assay. Protein extracts were separated on 10%–12% SDS-PAGE gels, transferred to PVDF membranes (Millipore), and probed with the following primary antibodies: Apaf-1 (1:1000, ABclonal, Wuhan, China), cleaved-caspase3 (1:500, Proteintech, Wuhan, China), Fis1 (1:2000, Proteintech, Wuhan, China), NBR1 (1:1000, Proteintech, Wuhan, China), Mfn2 (1:4000, Proteintech, Wuhan, China), LC3 (1:1000, ABclonal, Wuhan, China), HDAC1(1:2000, ABclonal, Wuhan, China), PINK1 (1:800, ABclonal, Wuhan, China), Parkin (1:2000, Proteintech, Wuhan, China), Beclin1 (1:2000, Proteintech, Wuhan, China), p62 (1:2000,Proteintech, Wuhan, China). And GAPDH (1:4000, Proteintech, Wuhan, China) was used as a loading control. The secondary antibody was incubated for 1 h at room temperature.

### TTC Staining

Brain tissue was quickly removed and frozen at −20 °C for 10 min; 1-mm-thick coronal sections were placed in 1.5% TTC and incubated at 37 °C for 15 min. Infarct volume was quantified using ImageJ software. Relative infarct volume was calculated as (contralateral volume minus ipsilateral non-infarct volume) divided by contralateral volume.

### TUNEL Staining

Paraffin-embedded brain sections (Ischemic penumbra of the cerebral cortex) were dehydrated, hydrated, and fixed according to the instructions of the TUNEL assay kit (KeyGEN BioTECH, China). TUNEL-positive cells were stained green, while normal cell nuclei were stained blue. Sections were observed under a fluorescence microscope.

### Immunofluorescence

Following deep anesthesia, mice were euthanized via transcardial perfusion with PBS. Ischemic penumbra of the cerebral cortex tissues was then collected, fixed in 4% paraformaldehyde, dehydrated in graded ethanol, and embedded in paraffin for sectioning (5 μm thickness). Coronal sections underwent antigen retrieval using citrate buffer (Beyotime, Jiangsu, China), followed by blocking with 10% goat serum in PBS for 1 h at room temperature. Sections were incubated overnight at 4 °C with the following primary antibodies diluted in PBS:LC3 and PINK1 (1:200, ABclonal, Wuhan, China); Beclin1, Apaf1, NeuN, P62 and NBR1 (1:100, Proteintech, Wuhan, China).After three 5-min washes with PBS, sections were incubated with Alexa Fluor-conjugated secondary antibodies (1:500) at 37 °C for 1 h. Nuclei were counterstained with DAPI (Solarbio, Beijing, China) for 10 min at room temperature. All steps included three PBS washes (5 min each). Images were acquired using a fluorescence microscope (400× magnification, Olympus).

### Grid Pharmacology and Molecular Docking

The targets of Oxymatrine were screened using the Swiss Target Prediction database (http://www.swisstargetprediction.ch/). All screened targets were converted into the UniProt ID format using the UniProt database (https://www.uniprot.org/).

### Prediction of Autophagy and Cerebral I/R Targets

We searched for “Autophagy” and “Cerebral ischemia–reperfusion” related targets in the GeneCardsdatabase (https://www.genecards.org/). The UniProt database (https://www.uniprot.org/) was used to convert all targets obtained from the above screening into the UniProt ID format.

### The Common Targets of Active Compounds and Targets Related to Autophagy and Cerebral I/R Overlap with Each Other

By identifying the common targets shared by autophagy and cerebral ischemia–reperfusion targets and the predicted Oxymatrine targets, a visual representation was shown in a Venn diagram using the VENN Website (http://bioinformatics.psb.ugent.be/webtools/Venn/).

### Molecular Docking

The crystal structures of the key targets were downloaded from the AlphaFold Protein Structure Database (https://alphafold.ebi.ac.uk/) and saved in PDB format. In AutoDockTools, water molecules are removed from the protein crystal structure, polar hydrogen atoms are added, and charges are added. Chemical constituents are in PDBQT format. In the grid box module, the distance of X, Y and Z coordinates is set to determine the active site of the protein. Molecular docking was performed in AutoDock Vina (https://autodock.scripps.edu/) to obtain the docking affinity (affinity, kcal/mol) between the active ingredient and the key target. The visualization of the molecular docking and its interactions was displayed in PyMOL (https://pymol.org/2/).

### Measurement of Oxidative Stress Markers (TAC, SOD, GSH/GSSG)

The levels of total antioxidant capacity (TAC), superoxide dismutase (SOD), and reduced/oxidized glutathione (GSH/GSSG) in brain tissues were measured using the following commercial assay kits: SOD Activity Kit (S0101S, Beyotime, China), TAC Detection Kit (BC1315, Solarbio, China), and GSH/GSSG Ratio Assay Kit (S0053, Beyotime, China). Briefly, the injured ischemic penumbra of the cerebral cortex was homogenized in ice-cold RIPA lysis buffer (P0013, Beyotime, China), and total protein concentrations were quantified via a BCA protein assay (BL521A, Biosharp, China) to normalize results to tissue weight (μg/mg). Tissue lysates were then incubated with kit-specific working solutions under manufacturer-specified conditions, followed by absorbance measurements at designated wavelengths using a BioTek Synergy H1 microplate reader. Final concentrations of MDA, TAC, SOD, and GSH/GSSG were calculated based on standard curves generated for each assay.

### Statistics Analysis

Data was collected from at least three independent experiments. Statistical analyses were performed using GraphPad Prism version 9.0 software (GraphPad, USA). Data were analyzed by one-way ANOVA with Tukey’s post hoc test for multi-group comparisons. Intergroup differences were assessed using unpaired Student’s *t* tests where appropriate. All values are expressed as mean ± SD. Statistical significance was set at *p* < 0.05.

## Results

### Oxymatrine Ameliorates Cerebral I/R Injury

**Fig. 1:**
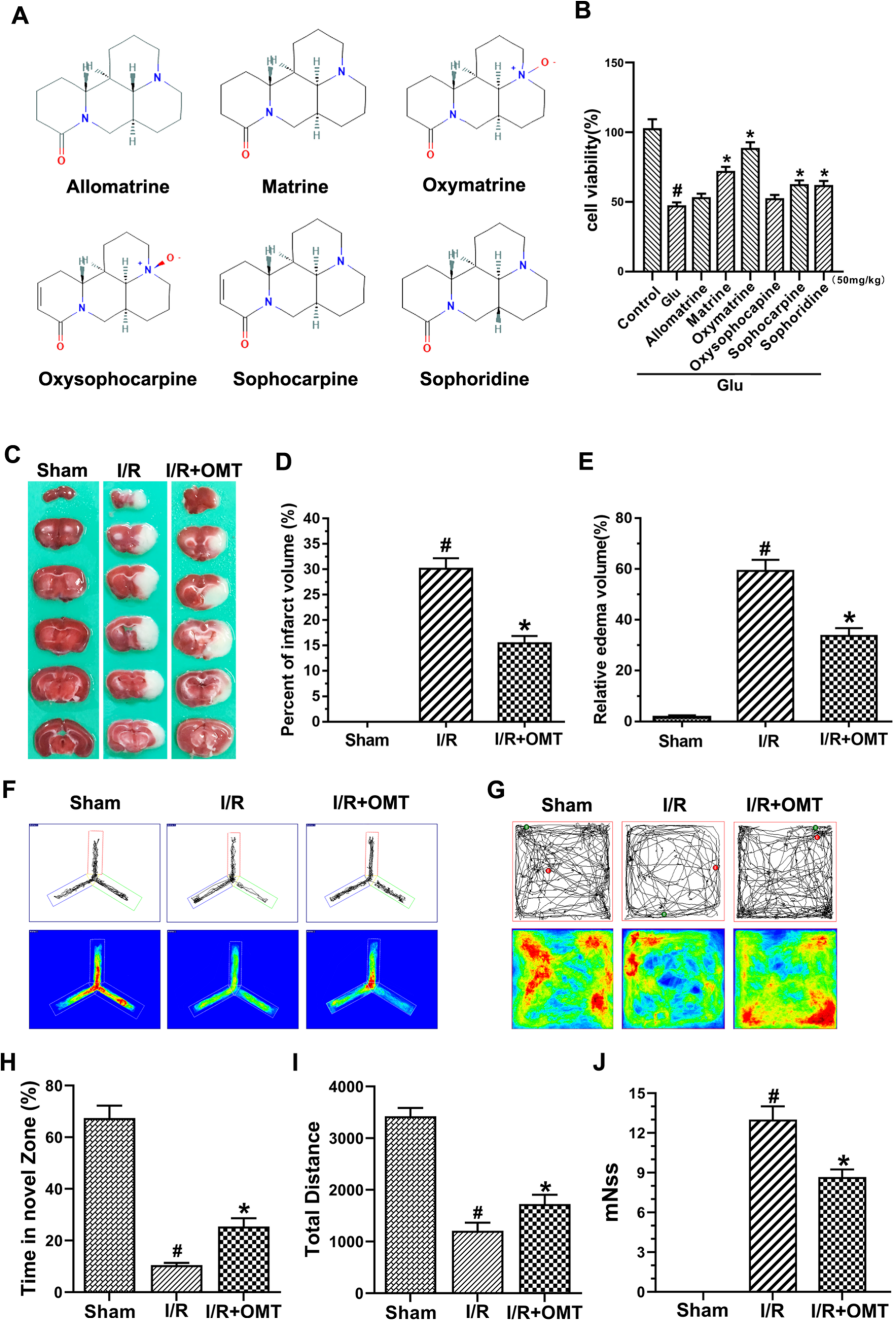
Oxymatrine ameliorates cerebral I/R injury. **A** Chemical formulas of six potential effective alkaloids. **B** Rescue effects on HT-22 cells in each group. **C** TTC staining in each group. **D** Quantification of the percent infarction volume. **E** Percentage of edema. **F**–**I** Behavioral tests. **J** Assessment of modified neurological severity score. ^#^*p* < 0.05 vs. Sham, *p < 0.05 vs. I/R

To demonstrate that oxymatrine is the most effective rescue drug, we screened active alkaloids based on the criteria from Yan et al. [23], selecting those with OB ≥ 30% and DL ≥ 0.18. Six potential effective alkaloids were identified: matrine, oxymatrine, allomatrine, sophocarpine, oxysophocarpine, and sophoridine. Cell experiments using the drug concentration tested by Yan et al. (50 mg/kg) showed that oxymatrine had the best protective effect [15], confirming it as the effective rescue drug (Fig. 1A, B).

To investigate the protective effect of oxymatrine on cerebral I/R injury, cerebral infarct volume, edema volume, and neurological deficit scores were measured. As anticipated, the I/R+OMT group exhibited reductions in cerebral infarct volume, edema volume, and deficit scores compared with the I/R group. The percentage of brain infarct volume in the I/R+OMT group significantly decreased from 30.08 ± 2.11% to 15.37 ± 1.48% (*p* < 0.05). The relative edema volume significantly decreased after oxymatrine treatment (Fig. 1C–E, J). Additionally, the mice’s motor and cognitive impairments were significantly improved after oxymatrine administration (Fig. 1F–I). The deficit scores also decreased after oxymatrine treatment (Fig. 1J). These results demonstrate that oxymatrine ameliorates cerebral I/R injury.

### Oxymatrine Ameliorates Apoptosis Induced by Cerebral I/R injury

**Fig. 2:**
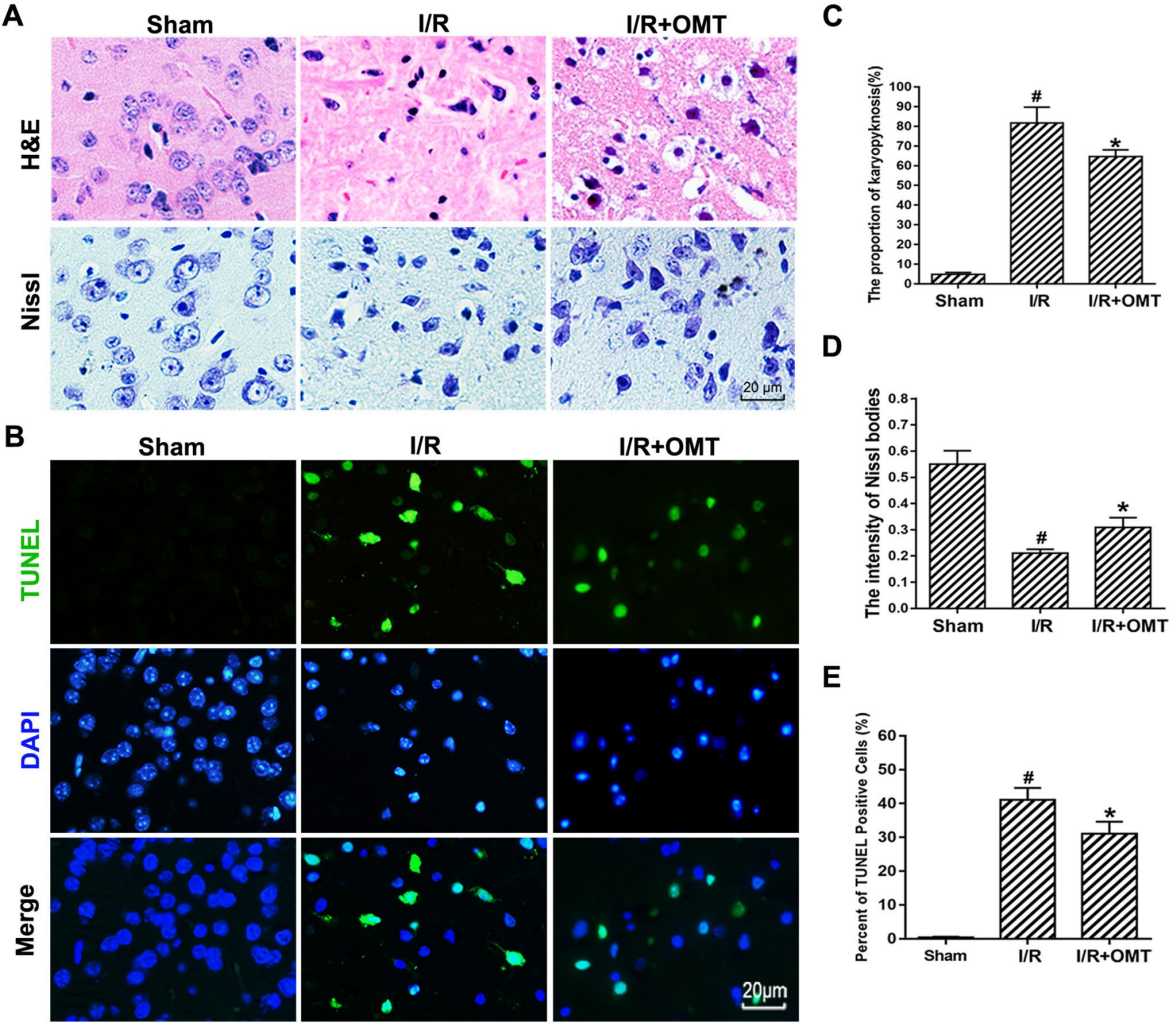
Oxymatrine reduces apoptosis induced by cerebral I/R in mice. **A** H&E staining and Nissl staining. **B** Representative images of TUNEL staining. **C** Quantitative summary of pyknotic cells from H&E staining. **D** The intensity of Nissl bodies. **E** Quantification of positive cells by TUNEL staining. ^#^*p* < 0.05 vs. Sham, *p < 0.05 vs. I/R

The effect of oxymatrine on morphological changes was also examined using H&E staining and Nissl staining. The cells were stained evenly and large with an abundant cytoplasmic compartment in the sham group (Fig. 2A, C, D). Conversely, most neurons in the I/R group exhibited shrunken cell bodies and were sparsely distributed. Moreover, the proportion of karyopyknosis was significantly higher, and the intensity of Nissl staining was significantly increased. However, this effect was significantly alleviated after induction with oxymatrine, which significantly decreases the proportion of karyopyknosis and reduces the intensity of Nissl bodies. These data demonstrate that the treatment with oxymatrine ameliorated pathological changes following cerebral I/R injury.

TUNEL staining was used to evaluate the effect of oxymatrine on apoptosis. As shown in Fig. 2B, E, I/R injury significantly increased the number of positive cells in the I/R group. Oxymatrine treatment significantly reduced the number of positive cells in the I/R+OMT group compared with the I/R group. Quantitative analysis showed that pre-treatment with oxymatrine reduced apoptotic cells by 43.37% (*p* < 0.05). In conclusion, oxymatrine can alleviate neuronal cell damage and cell apoptosis caused by brain I/R injury in mice.

### Oxymatrine Ameliorates Excessive Autophagy Induced by Cerebral I/R Injury

**Fig. 3:**
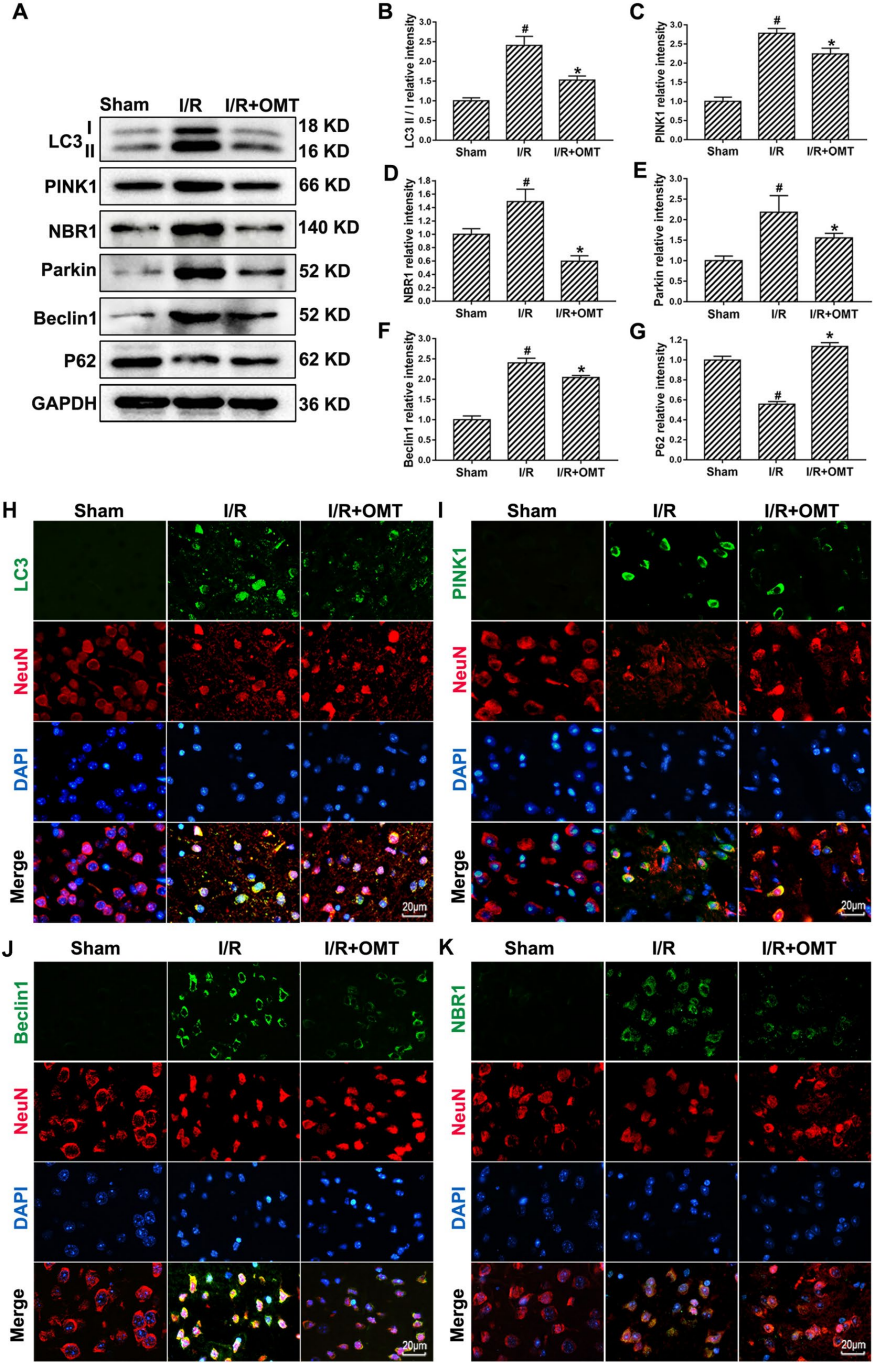
Oxymatrine ameliorates excessive autophagy induced by cerebral I/R in mice. **A** Western blotting results. **B**–**G** Western blotting analyses of LC3-II/I, PINK1, Parkin, NBR1, Beclin-1 and P62 in different groups when normalized to GAPDH. **H**–**K** Representative images showing double immunofluorescence staining for autophagy-related proteins (LC3, PINK1, Beclin1, NBR1) co-localized with neurons in different groups. Scale bar = 20 μm. ^#^*p* < 0.05 vs. Sham, ^*^p < 0.05 vs. I/R

The effect of oxymatrine on the autophagy signaling pathway after I/R injury was further investigated, and the changes in the levels of related proteins were detected by Western blotting and immunofluorescence. Compared with the sham group, the LC3-II/I ratio and the expression of PINK1, Parkin, Beclin-1, and NBR1 in the I/R group increased, while the level of P62 decreased. In contrast, the I/R+OMT group showed the opposite trend. Compared with the I/R group, the levels of key proteins promoting autophagy in the I/R+OMT group decreased (Fig. 3A–G). In contrast, the I/R+OMT group demonstrated a reversal of these trends. Specifically, oxymatrine treatment in the I/R+OMT group led to a decrease in the levels of key autophagy-promoting proteins. Moreover, the P62 protein, which inhibits autophagy, showed an increased level in the I/R+OMT group, suggesting that oxymatrine influenced the regulation of autophagy. Immunofluorescence analysis further confirmed these results, indicating that oxymatrine treatment reduced the expression of autophagy-related proteins induced by cerebral I/R injury. Immunofluorescence co-localization studies revealed that LC3, PINK1, Beclin-1, and NBR1 were co-expressed with the neuronal marker NeuN (Fig. 3H–K). This suggests that I/R injury induces excessive autophagy in neurons, and oxymatrine alleviates this excessive autophagy via the PINK1/Parkin pathway in mouse neurons.

### Oxymatrine Attenuates Glutamate-Induced HT22 Cell Injury by Suppressing Mitochondrial Apoptosis and Oxidative Stress

**Fig. 4:**
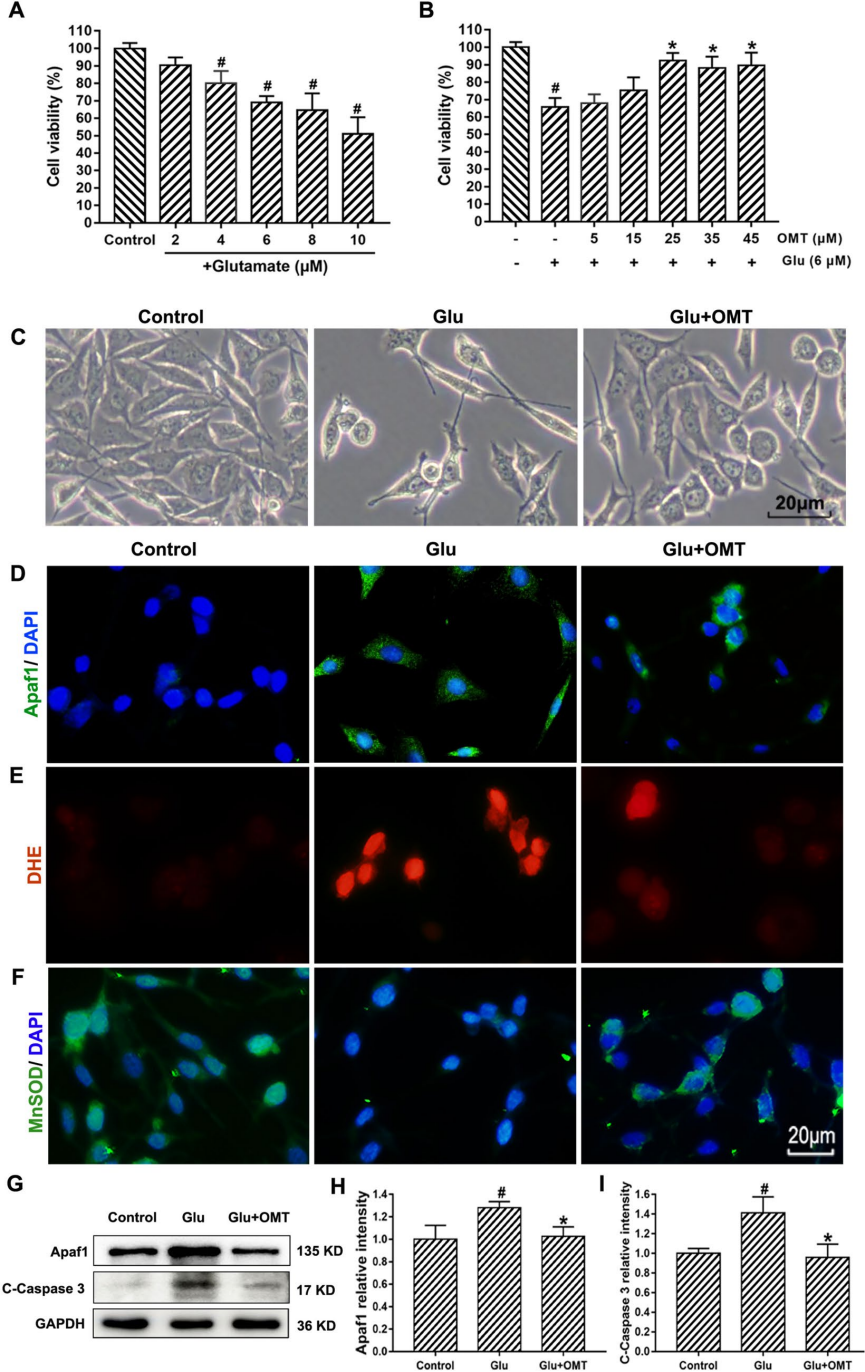
Oxymatrine attenuates glutamate-induced HT22 cell injury via dual mechanisms of mitochondrial apoptosis inhibition and ROS suppression. **A** Cell viability assay. HT22 cells were treated for 24 h with glutamate (2, 4, 6, 8, 10 μM). **B** Improvement of cell viability by various concentrations of oxymatrine (5–45 μM) in HT22 cells exposed to 6 μM glutamate for 24 h. **C** Light microscopic images of HT22 cells. Scale bar = 20 μm. **D** Immunofluorescence analysis of Apaf-1. Scale bar = 20 μm. **E** Superoxide radicals detected by DHE probe (red color) and **F** MnSOD detected using antibody (green color). Scale bar = 20 μm. **G** Western blotting of Apaf-1 and cleaved caspase3. **H**, **I** Semi-quantification of Apaf-1, total and cleaved caspase3 in different groups when normalized to GAPDH. ^#^*p* < 0.05 vs. Control, *p < 0.05 vs. Glu

To investigate the neuroprotective effect of oxymatrine against glutamate-induced cytotoxicity and oxidative stress in HT22 cells, a series of dose–response experiments were performed. As shown in Fig. 4A, cell viability decreased in a dose-dependent manner with increasing glutamate concentrations ranging from 2 μM to 10 μM. Specifically, 6 μM and 10 μM glutamate reduced cell viability to approximately 69% and 49%, respectively, compared to control. Based on these data, 6 μM glutamate was selected as the working concentration for subsequent experiments.

Next, to determine the optimal protective concentration of oxymatrine, HT22 cells were co-treated with 6 μM glutamate and varying concentrations of oxymatrine (5–45 μM). The greatest protective effect was observed at 25 μM, which was therefore selected as the working concentration for all following analyses (Fig. 4B). Morphological observations under microscopy further confirmed that oxymatrine treatment alleviated the characteristic cellular damage caused by glutamate exposure (Fig. 4C).

To explore whether oxymatrine attenuates mitochondrial apoptosis, we examined the expression and cellular localization of apoptotic protease activating factor-1 (Apaf-1). Immunofluorescence analysis revealed minimal Apaf-1-positive cells in the control group, while a marked increase was observed following 24-h glutamate exposure (Fig. 4D). Oxymatrine treatment significantly reduced the number of Apaf-1-positive cells in glutamate-challenged cultures. These findings were corroborated by Western blot results, which showed increased Apaf-1 protein levels after glutamate treatment and a marked reduction upon oxymatrine intervention. Additionally, we assessed caspase-3 activation as a downstream marker of mitochondria-mediated apoptosis. Glutamate exposure led to a robust increase in cleaved caspase-3 expression, without affecting total caspase-3 levels (Fig. 4G–I). Treatment with oxymatrine significantly downregulated cleaved caspase-3, further suggesting that oxymatrine mitigates glutamate-induced neuronal apoptosis via the mitochondrial pathway.

Given the role of reactive oxygen species (ROS) in mitochondrial dysfunction and apoptosis, we next evaluated ROS accumulation using the DHE fluorescent probe. Glutamate markedly elevated intracellular ROS levels compared to control, whereas oxymatrine treatment significantly suppressed this increase (Fig. 4E). To further elucidate oxymatrine’s antioxidative mechanism, we examined the expression of manganese superoxide dismutase (MnSOD), a key mitochondrial antioxidant enzyme. Immunofluorescence analysis demonstrated that glutamate reduced MnSOD-positive cells, while oxymatrine restored MnSOD immunoreactivity. Western blotting provided consistent results, showing a significant increase in MnSOD expression in the oxymatrine-treated group compared to glutamate alone (Fig. 4F). These findings suggest that oxymatrine’s neuroprotective effects are mediated, at least in part, through the attenuation of oxidative stress and enhancement of intrinsic antioxidant defenses.

### Oxymatrine Attenuates Glutamate-Induced HT22 Cell Injury by Regulating Mitochondrial Dynamics and PINK1/Parkin-Mediated Autophagy

**Fig. 5:**
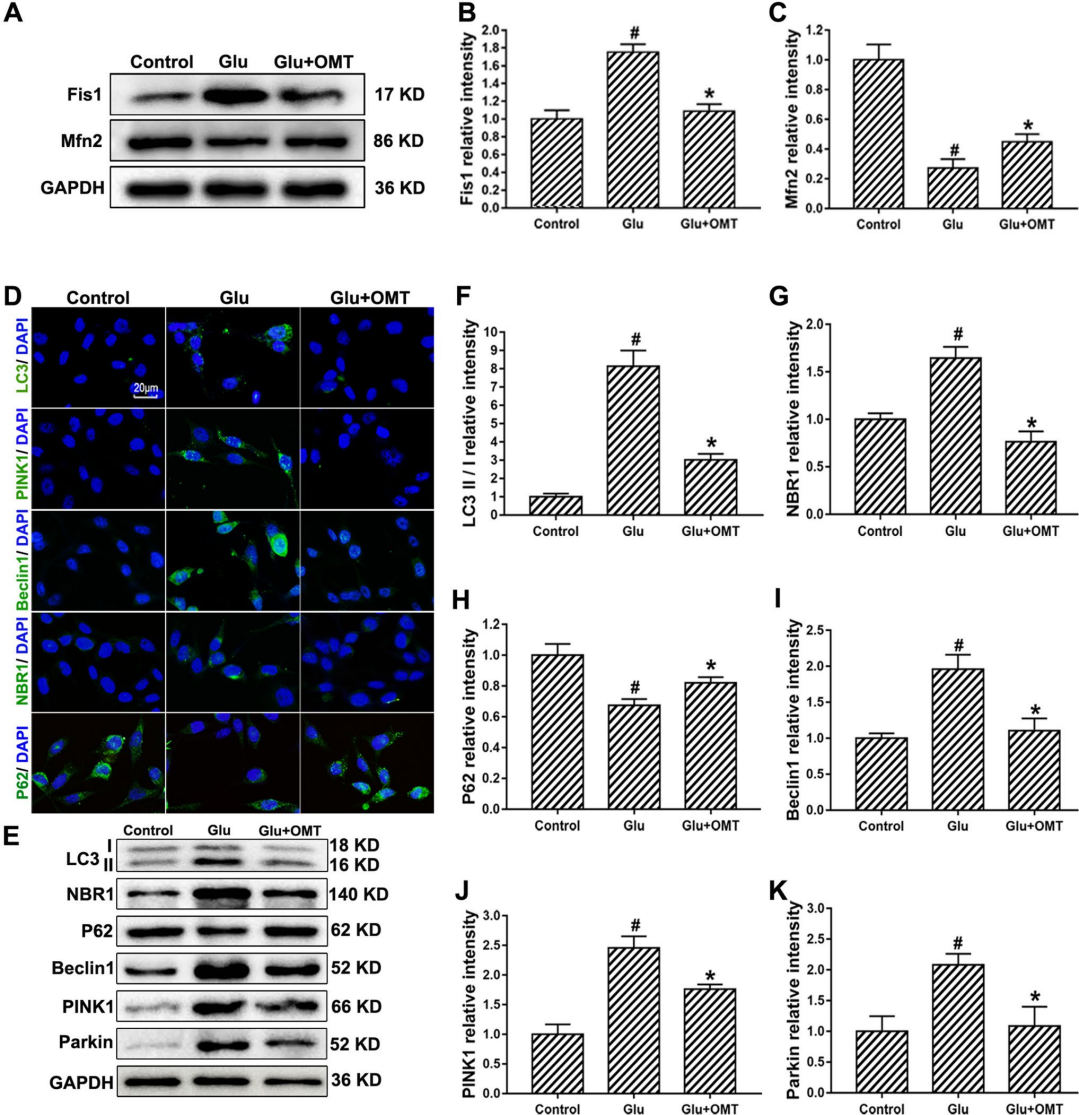
Oxymatrine restores mitochondrial homeostasis via coordinated modulation of fission/fusion balance and autophagy in glutamate-treated HT22 cells. **A** Western blotting for Fis1 and Mfn2. **B**, **C** Quantification of western blotting. **D** Immunofluorescence analysis showing the expression of LC3, PINK1, Beclin-1, NBR1 and P62. Scale bar = 20 μm. **E** Western blotting for LC3-II/I, PINK1, Beclin-1, NBR1 and P62. **F**–**K** Quantification of western blotting. ^#^*p* < 0.05 vs. Control, *p < 0.05 vs. Glu

Mitochondrial dynamics, defined by the balance between fission and fusion processes, are critical for maintaining cellular homeostasis and function. To assess whether oxymatrine influences mitochondrial dynamics in the context of glutamate-induced neuronal injury, we analyzed the expression of key regulatory proteins by western blotting in HT22 cells. Glutamate exposure significantly elevated the levels of the mitochondrial fission protein Fis1, while simultaneously reducing the expression of the fusion-related protein Mfn2, indicating a shift toward mitochondrial fragmentation. Interestingly, co-treatment with oxymatrine reversed this imbalance. Specifically, oxymatrine markedly suppressed the glutamate-induced increase in Fis1 levels, while restoring Mfn2 expression to levels closer to those observed in control cells (Fig. 5A–C). These findings suggest that oxymatrine mitigates glutamate-induced mitochondrial fragmentation and preserves mitochondrial dynamics, thereby contributing to cellular protection.

To further elucidate the role of oxymatrine in modulating autophagy, particularly through the PINK1/Parkin pathway, we examined the expression levels of multiple autophagy-related proteins using Immunofluorescence (Fig. 5D) and Western blotting (Fig. 5E–K). Following glutamate treatment, HT22 cells exhibited a substantial increase in the LC3-II/I ratio and elevated expression of PINK1, Parkin, Beclin-1, and NBR1, coupled with a notable reduction in P62 levels. These alterations are indicative of enhanced autophagy activity triggered by glutamate-induced mitochondrial stress.

Notably, oxymatrine intervention significantly reversed these glutamate-induced changes. Oxymatrine treatment reduced the LC3-II/I-a ratio and the expression of PINK1, Parkin, Beclin-1, and NBR1, while simultaneously restoring P62 levels. These results strongly support the notion that oxymatrine suppresses excessive autophagy via downregulation of the PINK1/Parkin signaling axis in glutamate-injured HT22 cells. Taken together, the data indicates that oxymatrine confers neuroprotective effects against glutamate-induced cytotoxicity by restoring mitochondrial dynamic balance and inhibiting pathologically elevated autophagy through modulation of the PINK1/Parkin pathway.

### Molecular Mechanisms Underlying Oxymatrine-Mediated Autophagy and I/R

**Fig. 6:**
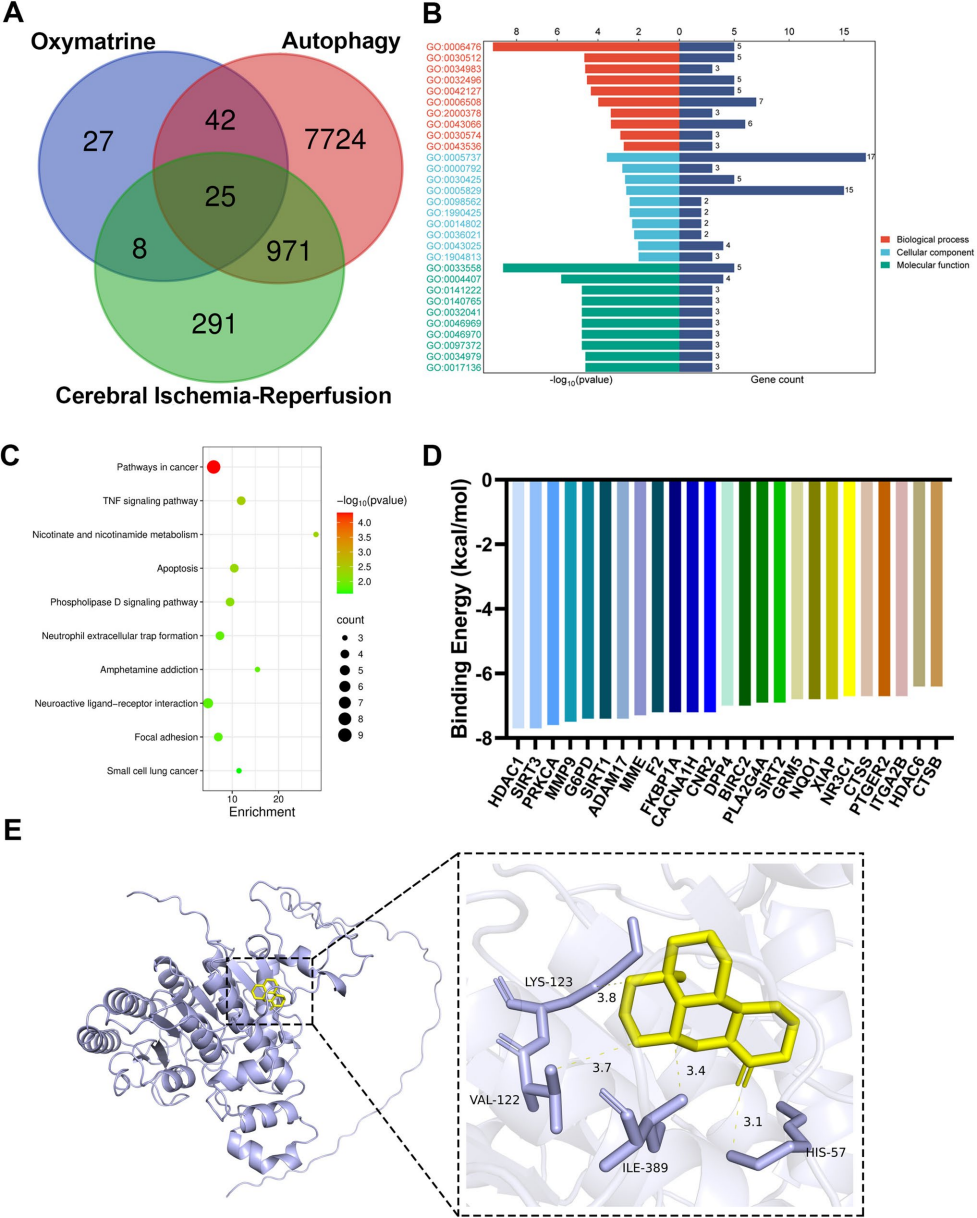
The regulation of Oxymatrine on HDAC1. **A** Venn diagram of Oxymatrine targets, autophagy targets, and cerebral I/R targets. **B**, **C** KEGG and GO enrichment analysis of 24 matching proteins. **D** Identification of 24 matching proteins, with HDAC1 having the lowest binding energy. Binding energy, calculated by molecular docking software, indicates the affinity between Oxymatrine and the predicted target protein (lower energy values indicate stronger binding affinity). **E** Binding sites and conformations of the complex formed by Oxymatrine and HDAC1

In order to understand the potential protein targets of Oxymatrine that may mediate the above autophagy and cerebral ischemia–reperfusion, we screened 100 potential targets of Oxymatrine through the Swiss Target-Prediction database. Based on the GeneCards v5.20 database, we obtained 8762 known targets related to autophagy and 1295 known targets related to cerebral ischemia–reperfusion; they match the target Oxymatrine composition as shown in a Venn diagram (Fig. 6A), and finally screened 25 agonistic protein targets that may have high affinity with Oxymatrine. Twenty-five targets of Oxymatrine that regulate autophagy and cerebral ischemia–reperfusion were mapped to the DAVID database for KEGG pathway enrichment analysis and biological function enrichment analysis. The results indicate a significant association of these genes related to the TNF signaling pathway, nicotinate and nicotinamide metabolism, neutrophil extracellular trap formation, and neuroactive ligand-receptor interaction (Fig. 6B). Biological processes such as protein deacetylation and negative regulation of the reactive oxygen species metabolic process participated in the regulation of Oxymatrine on autophagy and cerebral ischemia–reperfusion. Cellular components such as cytoplasm, heterochromatin, and dendrite are involved in the regulation of autophagy and cerebral ischemia–reperfusion by Oxymatrine. Molecular functions such as protein lysine deacetylase activity, histone deacetylase activity, histone H3K4 deacetylase activity, NAD-dependent, and histone H3K56 deacetylase activity, NAD-dependent and so on participate in the regulation of Oxymatrine on autophagy and cerebral ischemia–reperfusion (Fig. 6C).

Furthermore, the ways by which Oxymatrine might mediate cerebral ischemia–reperfusion protein targets were simulated by AutoDock vina software. The results showed that the binding energy of Oxymatrine to HDAC1 was the lowest (Fig. 6D). As for HDAC1, Oxymatrine occupies the active site of HDAC1 by forming a hydrogen bond with HIS57 and it has hydrophobic interactions with VAL122, LYS123, and ILE389 (Fig. 6E).

### Inhibition of HDAC1 Attenuates the Neuroprotective Effects of Oxymatrine in I/R Injury

**Fig. 7:**
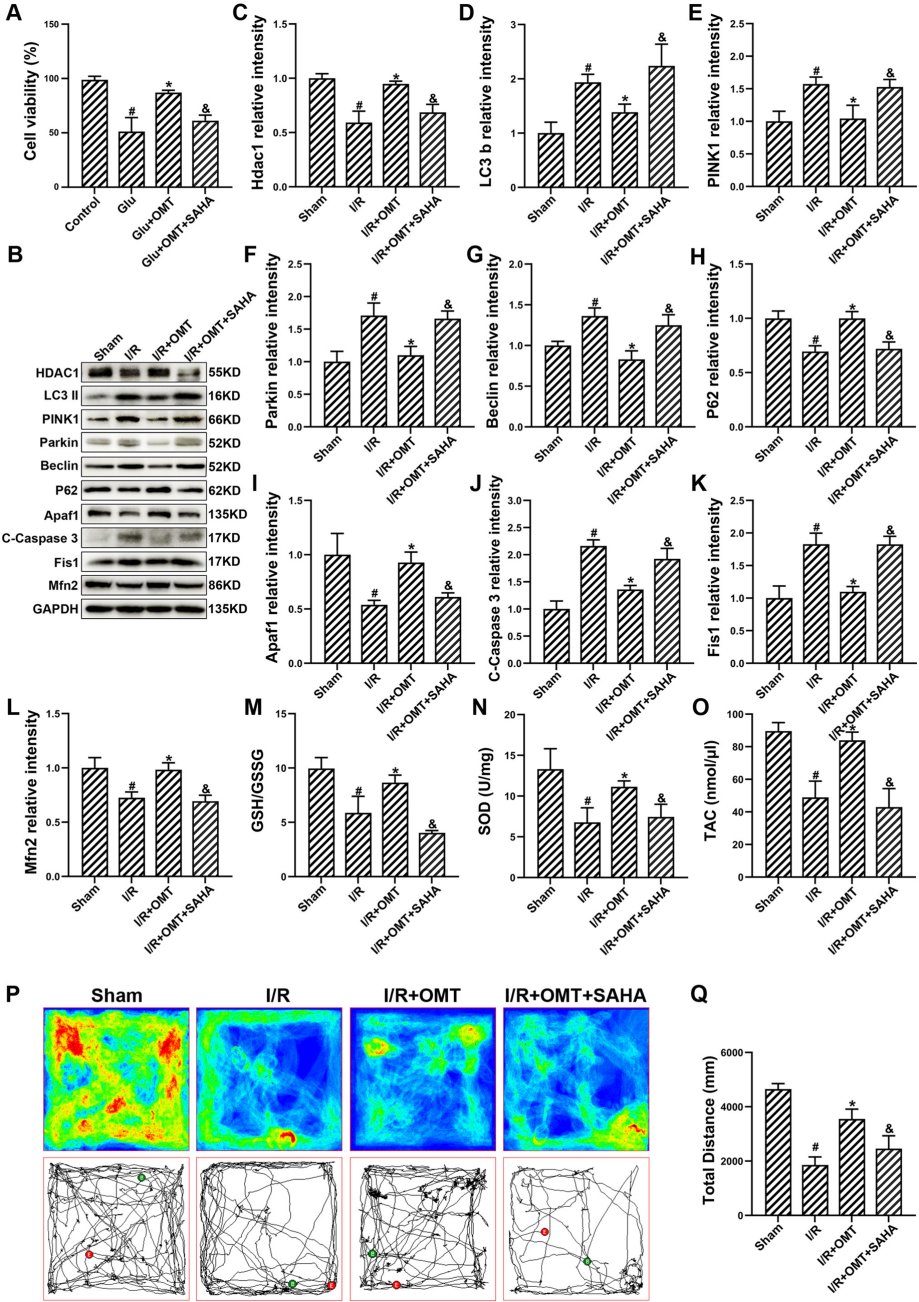
OMT mediates neuroprotection in cerebral I/R injury via HDAC1**.** **A** Cell viability in HT22 cells. **B**. Representative Western blotting for HDAC1, LC3-B, PINK1, Parkin, Beclin-1, P62, Apaf-1, cleaved caspase-3, Fis1, and Mfn2. **C–L** Quantification of Western blot bands for mitochondrial dynamics and autophagy markers. **M** GSH/GSSG ratio in brain tissue. **N** SOD activity in brain tissues. **O** Total antioxidant capacity (TAC) levels in brain tissues. **P** Tracking of mouse movement in the open field test. **Q** Total distance traveled in the open field test. ^#^*p* < 0.05 vs. Control or Sham, *p < 0.05 vs. Glu or I/R, ^&^*p* < 0.05 vs. I/R+OMT

In this study, molecular docking analysis identified HDAC1 as a potential target of OMT in the context of cerebral I/R injury. To validate the role of HDAC1 in mediating the neuroprotective effects of OMT, we utilized SAHA, an HDAC1 inhibitor, to investigate its impact on OMT-induced neuroprotection. Cell viability was assessed under various treatments, including control, glutamate (Glu), glutamate + OMT, and glutamate + OMT + SAHA groups. As shown in Fig. 7A, OMT treatment significantly improved cell viability in glutamate-exposed HT22 cells, while the combined treatment with SAHA partially reversed this effect. Western blot analysis revealed significant changes in key proteins associated with mitochondrial dynamics, autophagy, and apoptosis. In the I/R+OMT group, the expressions of HDAC1, LC3-B, PINK1, Parkin, Beclin-1, P62, Apaf-1, cleaved caspase-3, Fis1, and Mfn2 were notably altered compared to the I/R group (Fig. 7B). The addition of SAHA (I/R+OMT+SAHA) reversed some of these changes, indicating that HDAC1 inhibition negatively affects the neuroprotective actions of OMT. Quantitative analysis of the protein bands for these markers (Fig. 7C-L) confirmed that oxymatrine treatment reduced the expression of pro-apoptotic proteins (Apaf-1, cleaved caspase-3) and autophagy markers (PINK1, Parkin, Beclin-1, NBR1), while promoting Mfn2. In contrast, the co-treatment with SAHA significantly reversed these effects, suggesting that the inhibition of HDAC1 attenuates the neuroprotective effects of OMT by disrupting mitochondrial dynamics and autophagy processes.

To assess the role of oxidative stress, we measured key antioxidant markers including the GSH/GSSG ratio, superoxide dismutase (SOD), and total antioxidant capacity (TAC). As shown in Fig. 7M–O, oxymatrine treatment increased the levels of GSH/GSSG, SOD, and TAC compared to the I/R group. However, SAHA co-treatment reduced the levels of these antioxidants, further supporting the hypothesis that HDAC1 inhibition impairs the antioxidative effects of OMT. Behavioral recovery was evaluated using the open field test, where Fig. 7P–Q shows the tracking and total distance traveled of mice in the sham, I/R, I/R+OMT, and I/R+OMT+SAHA groups. The I/R group exhibited significant deficits in locomotion and anxiety behavior, while OMT treatment improved both parameters. However, the combined treatment with SAHA reversed these behavioral improvements, suggesting that HDAC1 inhibition impedes the neuroprotective effects of OMT on functional recovery.

## Discussion

This study provides the first evidence that OMT exerts neuroprotective effects in I/R injury by targeting HDAC1, simultaneously regulating mitochondrial dynamics and autophagy. This finding not only expands the understanding of the neuroprotective mechanisms of traditional Chinese medicine components but also provides a novel theoretical basis for multi-target intervention strategies in I/R injury.

As the main active alkaloid of Sophora flavescens, the neuroprotective potential of OMT has been preliminarily validated in previous studies, including the inhibition of inflammation, reduction of oxidative stress, and improvement of microvascular integrity [13, 24, 25]. The core breakthrough of this study is revealing that OMT mediates epigenetic regulation through HDAC1, leading to the coordinated regulation of mitochondrial fission/fusion (Fis1/Mfn2) and PINK1/Parkin-dependent autophagy. This mechanism significantly differs from existing studies. In current neuroprotective strategies, such as reperfusion therapy, intravenous thrombolysis is an important treatment for acute ischemic stroke [26, 27]. However, its narrow therapeutic window, strict eligibility criteria, and low recanalization rates result in a high incidence of complications, including hemorrhagic transformation. Endovascular thrombectomy is recommended by guidelines, but some patients, even with treatment within the therapeutic window, still show poor prognosis [28, 29]. These conventional treatments primarily focus on restoring blood flow but fail to effectively address the complex biochemical responses induced by I/R.

In terms of pharmacological intervention, most current neuroprotective drugs target a single pathway. For example, antioxidants like edaravone reduce damage by scavenging free radicals [30, 31], anti-inflammatory drugs like metformin focus on inhibiting inflammatory factors [32], and autophagy modulators such as ginsenoside Rg1 regulate autophagic activity through the mTOR pathway [33]. In contrast, OMT targets HDAC1 as a molecular hub, simultaneously intervening in mitochondrial homeostasis and autophagic flux, a level of systemic regulation difficult to achieve with current single-target strategies. Regarding the mechanistic innovation of HDAC1, most existing studies focus on its role in chromatin remodeling to regulate the transcription of inflammation-related genes (such as IL-1β, TNF-α) or its involvement in cell cycle regulation [34–36]. Studies have found that abnormal HDAC1 function can promote blood–brain barrier damage by disrupting tight junction proteins [34]. However, this study is the first to discover that HDAC1 serves as an upstream regulator of the mitochondrial-autophagy axis, coordinating the Fis1/Mfn2 and PINK1/Parkin pathways through epigenetic modification. Specifically, the HDAC1 inhibitor SAHA not only reversed OMT’s downregulation of the autophagy marker LC3-II/I but also blocked its restoring effect on the mitochondrial fusion protein Mfn2.

This discovery significantly expands the known functions of HDAC1, transforming it from a mere inflammation regulator to a key epigenetic switch that integrates mitochondrial function and autophagy, providing a new paradigm for understanding the multidimensional role of epigenetic regulation in stress-related cellular responses. Notably, although bioinformatics predictions and molecular docking show strong binding affinity between OMT and HDAC1 (with the lowest binding energy), and SAHA reverses OMT’s protective effect, this study has not yet directly validated the necessity of HDAC1 through siRNA-mediated knockdown experiments. Neither HDAC1 activity assays nor pull-down experiments have been performed to directly validate the binding, and these experiments will be a focus of future research to strengthen the evidence chain for HDAC1 as a functional target of OMT.

The innovation of this study lies in confirming that the regulation of the PINK1/Parkin pathway is epigenetically controlled by HDAC1. It is known that mitochondrial autophagy mediated by the PINK1/Parkin pathway is primarily activated through a kinase cascade reaction. Early in brain ischemia, mitochondrial dysfunction triggers a series of changes, with the PINK1/Parkin pathway playing a key role in this process [37, 38]. For example, changes in the protein levels associated with this pathway and mitochondrial autophagy dysfunction have been detected in the hippocampus of patients with mild cognitive impairment and relevant animal models [39–41]. However, this study found that OMT downregulates the expression levels of PINK1/Parkin by stimulating HDAC1, suggesting that transcriptional regulation of PINK1/Parkin may serve as an early adaptive response under stress conditions.This indicates that cells employ multi-level mitochondrial quality control strategies in response to I/R injury, and the HDAC1-PINK1/Parkin axis targeted by OMT represents an upstream regulatory mechanism that has yet to be fully recognized.

From a therapeutic strategy perspective, the multi-target synergistic effect of OMT overcomes the limitations of current single-pathway interventions. The pathological network of brain I/R injury involves multiple interconnected pathways, including oxidative stress, inflammation, mitochondrial damage, and autophagy dysregulation [42–45]. Single-target interventions often have limited efficacy due to compensatory mechanisms. For instance, solely inhibiting autophagy may result in inadequate clearance of damaged mitochondria, while excessive promotion of mitochondrial fusion may impair energy metabolism efficiency [46–48]. In this study, OMT synchronously calibrates mitochondrial fission/fusion balance and autophagic activity via HDAC1. This mechanism prevents excessive ROS release due to mitochondrial fragmentation while avoiding excessive autophagy-induced neuronal degradation. Compared to strategies that solely regulate autophagy or mitochondrial dynamics (such as Rg1 or Schisandrol A) [33, 49], OMT exhibits superior capability in maintaining cellular homeostasis. Additionally, the antioxidant and anti-apoptotic effects of OMT further enhance its multidimensional protective properties, offering more upstream intervention value compared to edaravone’s antioxidant and anti-inflammatory effects.

This study has several limitations: First, the validation of HDAC1 as a target of OMT requires further investigation. Although molecular docking and SAHA blockade experiments provide indirect evidence, functional rescue experiments after siRNA-mediated knockdown are lacking. Additionally, the interaction between OMT and HDAC1 has not been confirmed through HDAC1 activity assays or direct binding experiments (such as surface plasmon resonance), which will be a key focus of future research. Second, direct observation of mitochondrial morphology and autophagic flux is insufficient; current results primarily rely on protein-level detection. The absence of MitoTracker staining or transmission electron microscopy (TEM) for observing mitochondrial ultrastructural changes, combined with the lack of dynamic tracking of autophagic flux using the mRFP-GFP-LC3 dual-tagging system, may impede intuitive interpretation of mitochondrial-autophagy interactions. Third, the regional consistency between in vitro and in vivo experiments needs to be improved. While the HT22 hippocampal neuronal model was used in vitro, the in vivo study did not specifically analyze pathological changes and molecular mechanisms in hippocampal neurons, making it difficult to exclude brain region-specific effects.

## Conclusions

**Fig. 8:**
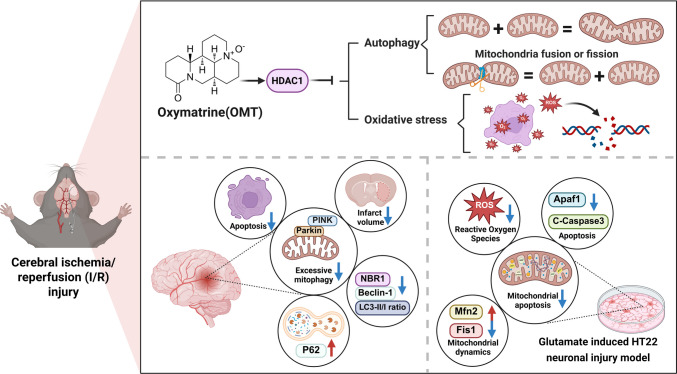
Mechanistic illustration of OMT-mediated neuroprotection in cerebral I/R injury. OMT upregulates HDAC1, which suppresses excessive autophagy by inhibiting the PINK1/Parkin pathway. This regulatory mechanism restores mitochondrial dynamics by balancing fission and fusion, thereby promoting improved mitochondrial homeostasis. As a result, oxidative stress is reduced, and neuronal viability is significantly enhanced. The neuroprotective effects of OMT were validated in both an in vivo mouse I/R injury model and an in vitro glutamate-induced HT22 neuronal injury model. These findings underscore the potential of OMT as a promising agent for the management of cerebral I/R injury

In this study, in vivo and in vitro models, including the MCAO model and glutamate-exposed HT22 hippocampal neurons, were used alongside bioinformatics and molecular docking. The results show that OMT provides neuroprotection by targeting HDAC1 (Fig. 8). It significantly reduced infarct size, improved neurological function, and restored mitochondrial dynamics by regulating Fis1 and Mfn2. OMT also inhibited excessive autophagy through the PINK1/Parkin pathway, as well as mitochondrial apoptosis and ROS accumulation, demonstrating antioxidant effects. These effects were reversed by the HDAC1 inhibitor SAHA. This study offers new insights and potential therapies for ischemic stroke treatment.

Mechanistic illustration of OMT-mediated neuroprotection in cerebral I/R injury. OMT upregulates HDAC1, which suppresses excessive autophagy by inhibiting the PINK1/Parkin pathway. This regulatory mechanism restores mitochondrial dynamics by balancing fission and fusion, thereby promoting improved mitochondrial homeostasis. As a result, oxidative stress is reduced, and neuronal viability is significantly enhanced. The neuroprotective effects of OMT were validated in both an in vivo mouse I/R injury model and an in vitro glutamate-induced HT22 neuronal injury model. These findings underscore the potential of OMT as a promising agent for the management of cerebral I/R injury

## Data Availability

Data will be made available on request.
